# Effect of Phase Structure on the Viscoelasticity and Mechanical Properties of Isotactic Polypropylene Multicomponents Polymerized with Non-Conjugated α,ω-Diene

**DOI:** 10.3390/polym16192715

**Published:** 2024-09-25

**Authors:** Songmei Zhao, Jin-Yong Dong, Yawei Qin, Chuanzhuang Zhao, Yuan Yu, Weili Liu

**Affiliations:** 1 Institute of New Materials and Advanced Manufacturing, Beijing Academy of Science and Technology, Beijing 100089, China; 2 CAS Key Laboratory of Engineering Plastics, Institute of Chemistry, Chinese Academy of Sciences, Beijing 100190, China; jydong@iccas.ac.cn (J.-Y.D.);; 3 University of Chinese Academy of Sciences, Beijing 100049, China; 4 School of Materials Science & Chemical Engineering, Key Laboratory of Impact and Safety Engineering, Ministry of Education, Ningbo University, Ningbo 315211, China

**Keywords:** crosslinking, iPP/EPR in-reactor alloys, high EPR content, phase structure, mechanical properties

## Abstract

Increasing of rubber content in isotactic polypropylene/ethylene–propylene rubber (iPP/EPR) alloys can extend the applications of this kind of polyolefin. The EPR content and phase structure of isotactic polypropylene multicomponents have great effect on the viscoelasticity and mechanical properties. iPP/EPR in-reactor alloys with a high EPR content were obtained through the in situ crosslinking of EPR chains with α,ω-diene. The morphological observation results indicate that the crosslinked iPP/EPR in-reactor alloys have a good spherical shape with clean and rough external surfaces. The high EPR content is finely dispersed in the crosslinked iPP/EPR alloys in areas ranging in size from tens of nanometers to several micrometers, which implies that a sufficient crosslinking degree of EPR chains can effectively prevent their aggregation and restrict macro-phase separation. The rheological results show a clear plateau in the terminal region, which reveals an entangled polymer chain network in the crosslinked iPP/EPR alloys. The well-dispersed EPR and the bi-continuous phase structure have a great effect on the mechanical properties of the isotactic polypropylene multicomponent which were assessed.

## 1. Introduction

Isotactic polypropylene (iPP) is replacing traditional and/or engineering plastics in automobiles and appliances because of its low cost, low density, excellent chemical durance, high tensile modulus and stiffness, and superior heat resistance [1,2,3]. However, iPP displays poor impact strength at low temperatures, which restricts its applications in some fields. Traditionally, one or several kinds of elastomer material are blended into iPP through melt or solvent blending [4,5,6,7,8]. With the development of in-reactor alloy technology, it has become easy to combine elastomers with iPP to obtain high-performance polyolefin materials through multi-reactor processes, which are easy to control and can be continuously performed on a large scale [9]. Compared to polyethylene thermoplastic elastomer, polypropylene thermoplastic elastomer has a higher melting point and increased strength and rigidity, which means that it can be more widely applied. The incorporation of EPR into iPP can entangle the polymer chains, and the elasticity and toughness of these materials can also be enhanced to some extent [10]. Using a Ziegler–Natta catalyst on an isotactic polypropylene/ethylene–propylene rubber (iPP/EPR) thermoplastic elastomer (i.e., TPO, in which the mass fraction of EPR is more than 50 wt.%) is a technological innovation in the industry [11]. However, increasing the EPR content in in-reactor alloys with a controllable particle morphology remains a challenge.

Some iPP in-reactor alloys have been obtained and studied using multiphase polymerization, such as iPP/EPR [4,12], polypropylene/ethylene-octane copolymers (PP/POEs) [13], isotactic polypropylene/poly(ethylene-co-octene) (iPP/PEOc) [14,15], and isotactic polypropylene/isotactic polybutene-1 (iPP/iPB) [16]. Du [17] and Zhou et al. [18] studied the growth mechanisms and pristine basic morphologies of polyolefin particles in iPP/PEOc and iPP/EPR alloys, which were synthesized using a Ziegler–Natta/metallocene hybrid catalyst and a state-of-the-art MgCl_2_/TiCl_4_-supported catalyst, respectively. The authors found that these iPP in-reactor alloys displayed a multiscale structure and self-similar hole-filling growth mechanisms. However, the EPR content in these alloys is usually lower than 35 wt.%. With an increase in EPR content, the EPR phase becomes aggregated and can flow into the interspace or surface of the alloys. For polymerization, Fu et al. [19,20] studied the effect of combined external electron donor diphenyldimethoxysilane/dicyclopentyldimethoxysilane (DDS/D-donor) mixtures and a multistage sequential polymerization process on the structure, morphology, and properties of iPP/EPR in-reactor alloys. They found that the molecular weight of iPP and the structure uniformity of multiblock poly(ethylene-co-propylene) increased as the amount of D-donor in the DDS/D-donor mixtures increased. The toughest iPP/EPR in-reactor alloy was obtained with an optimum feed of DDS and D-donor at a molar mass ratio of 1:3.

In this system, the cyclic switch in the first propylene homo-polymerization and second ethylene–propylene copolymerization stages cannot change the EPR content in iPP/EPR alloys, but it can reduce the EPR phase size and distribution, thus improving the impact strength and the bending modulus. In addition to the aforementioned studies, the effects of morphology, crystallization behavior, mechanical properties, and structural changes, including the degradation and crosslinking of iPP/EPR alloys (also called impact polypropylene copolymers), need to be explored [3,21,22,23]. In these iPP/EPR in-reactor alloys, the elastomer is generated at the iPP particle interface and interspaces [24]. The aggregation of the EPR phase usually occurs during polymerization with an increase in elastomer content. On the one hand, aggregated elastomers hinder the flow of the monomers into the iPP particles, which greatly restricts the incorporation of high-content rubber into the alloys. On the other hand, a polymeric elastomer may continue gathering and fleeing to the surfaces of the alloys, which causes sticking in the polymerization process and deteriorates the products. Some results, such as molecular structure, local dynamics, condensed phase separation, and crystallization, have been reported, but further studies are required, not only to improve the design of advanced polyolefin, but also to reveal the structure–property relationships [14,25,26,27,28,29].

In this study, by introducing a small amount of non-conjugated α,ω-diene as a crosslinking agent during the copolymerization process, a series of crosslinked iPP/EPR in-reactor alloys with a high elastomer content were prepared. The microstructure, morphology, rheology, and mechanical properties of the crosslinked iPP/EPR in-reactor alloys were studied in detail.

## 2. Experimental Design

### 2.1. Material Polymerization

The iPP/EPR in-reactor alloys were synthesized through two one-pot steps, involving in situ polymerization with a Ziegler–Natta catalyst, i.e., a MgCl_2_/TiCl_4_ catalyst using 9,9-bis(methoxymethyl)fluorine (BMMF) as an internal electron donor. During the first stage, the propylene was homo-polymerized for 30 min under 60 °C, which was initiated with the Ziegler–Natta catalyst. After the homo-polymerization stage, the solvent and the other liquid substances were removed by vacuum evaporation; only the iPP particles remained in the steel autoclave reactor. Then, the mixed ethylene and propylene (~1/1) were fed into the reactor to conduct the second stage of polymerization at 90 °C. At the same time, a small amount (0–1.35 mmol) of non-conjugated α,ω-diene was added for the simultaneous crosslinking of the EPR chains during this stage. After copolymerization, the samples were treated and terminated with water steam, and, finally, a series of high-EPR-content iPP/EPR alloys were obtained. In addition, the primary iPP and pristine iPP/EPR alloys without crosslinking were also polymerized under the same conditions for comparison, which are denoted as iPP and iPP/EPR-1, respectively.

### 2.2. Material Characterization

*Component Contents*: The contents of EPR in the iPP/EPR alloys were determined through the separation of xylene. The iPP/EPR alloys (*m*_0_) were dissolved in boiling xylene for 2 h, and then cooled to room temperature. A clear solution of cold methanol and solvent was deposited and evaporated, and the undissolved part was dried at 60 °C in an oven. The dissolved EPR (*m*_1_) and iPP parts (*m*_2_) were obtained. The dissolved EPR content (*w_sol_*) was calculated using this equation:

*w_sol_* (wt.%)= *m*_1_/*m*_0_ × 100%

The gel content (*w_gel_*) was determined via solvent extraction, where the alloys were first boiled in xylene for 12 h, and then further extracted for another 6 h. All the samples were dried at 60 °C for more than 24 h. In this paper, *w_sol_* signifies the component that was soluble in boiling heptane, *w_gel_* signifies the insoluble part after xylene extraction, and *w_EPR_* denotes the total EPR content, which is the combined random and crosslinked EPR content in the iPP/EPR alloys. *w_EPR_* was calculated by combing *w_sol_* and *w_gel_*.

The thermal properties of the iPP/EPR alloys were determined using a Perkin-Elmer Pyris Diamond differential scanning calorimeter (DSC). The specimens were heated to 200 °C for 5 min, then cooled back to 30 °C, and then reheated to 200 °C at a ramp rate of 10 °C/min. The reheating curves were extrapolated to understand the thermal properties and crystallinity of these alloys. Fourier-transform infrared spectroscopy (FTIR) was conducted using a Nicolet 6700 instrument with an ATR accessory, and the wavelength ranged from 650 to 4000 cm^−1^ with a resolution of 4 cm^−1^. The molar mass of ethylene content in the EPR was calculated from the characteristic FTIR absorption band by the area of 1376 cm^−1^ (A_1376_) and 1459 cm^−1^ (A_1459_). Nuclear magnetic resonance (NMR) spectra were recorded on a Bruker 500 M spectrometer at 110 °C using o-dichlorobenzene-d_4_ as a solvent.

The morphology of the crosslinked iPP/EPR alloys was observed with a JEOL (JSM 6700F) scanning electron microscope (SEM) with an operating voltage of 5.0 kV. The samples were coated with platinum before observation. The internal and phase dispersions of the EPR domains in the plain and crosslinked iPP/EPR alloys were observed with a high-resolution atomic force microscope (AFM). The specimens were polished at −90 °C using a Leica EM UC6 ultra-microtome to obtain a smooth surface.

Dynamic storage (*G*′) and loss modulus (*G*″) for the iPP/EPR alloys were measured in small-amplitude oscillatory shear flow using the TA rheometer with parallel plates, and the sample thickness was 1.0 mm. Dynamic frequency sweeps were conducted at a constant temperature of 200 °C with strain amplitude of 1.25% under gaseous nitrogen atmosphere, and the angle frequency (*w*) ranged from 0.01 to 500 rad/s. Dynamic mechanical analysis (DMA) was performed on a DMA Q800 (TA, USA). The specimen size was approximately 18.0 × 12.5 × 1.6 mm^3^, then they were tested in a single-cantilever model with a frequency (*f*) of 1.0 Hz. The samples were tested from −130 °C to 130 °C with a heating rate of 3 °C/min under a nitrogen atmosphere. The mechanical properties of iPP/EPR alloys were measured with an Instron 3365 instrument according to the standards GB/T 9341–2000 and GB/T 2040.2–2006, respectively. Impact strength tests were carried out on an XJC-250 impact strength tester according to GB/T 1843–1996. All specimens were prepared by extrusion molding using a Haake Minijet operated at 200 °C and 8 × 10^7^ Pa, with a molder temperature of 40 °C.

## 3. Results and Discussion

### 3.1. Synthesis of Crosslinked iPP/EPR Alloys

**Figure 1:**
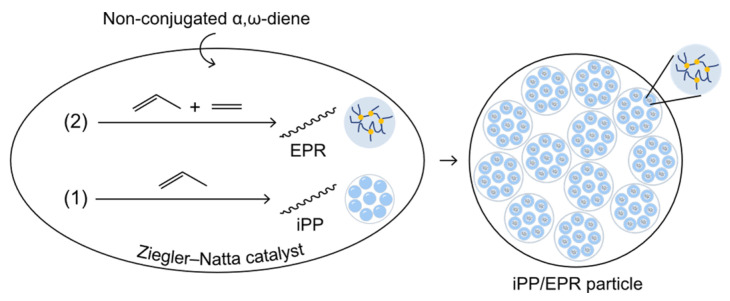
Synthetization and particle growth of iPP/EPR, with enlarged crosslinked EPR chains and bonds.

Synthetization and particle growth of iPP/EPR alloys was shown in Figure 1. iPP/EPR in-reactor alloys with a high EPR content were obtained through the simultaneous crosslinking of EPR chains with non-conjugated α,ω-diene. The polymerization was carried out using a Ziegler−Natta catalyst, and the synthesis of iPP/EPR in-reactor alloys with EPR chain crosslinking is shown in Figure 1. During the synthetization process, the first step was propylene homo-polymerization, and the second step was ethylene–propylene copolymerization. During the second stage, a crosslinker agent, i.e., dihexanoxene dichlorosilane (DHDCS), was added to launch the crosslinking of EPR chains simultaneously. In a typical polymerization reaction, iPP particles are first generated in the first propylene homo-polymerization stage, following the replication mechanism of the Ziegler−Natta catalyst with spherical morphology. There are pores and interspaces between the primary and secondary iPP particles, which can allow the ethylene and propylene copolymer to polymerize and fill these spaces [30]. In the next stage, a mixed ethylene/propylene gas and a few DHDCS molecules were fed into the reactor for ethylene–propylene copolymerization that simultaneously occurred with the EPR chain branching/crosslinking. Finally, the spherical crosslinked iPP/EPR in-reactor alloys were obtained.

Polymerization of the iPP and iPP/EPR alloys is displayed in Table 1. During this copolymerization process, a sufficient number of crosslinked EPR chains in the iPP/EPR particles can effectively inhibit the flowing of EPR from the alloys to iPP interspaces or iPP holes. In this case, the EPR phase, which are formed from mobile droplets with segregation to stabilize discrete particles through the adhesion of catalyst fragments, are evenly dispersed and embodied into iPP mesh particles. Therefore, the EPR content in the alloys can be efficiently increased and accompanied with fine dispersion in the iPP matrix. In this case, the EPR content in the iPP/EPR alloys was increased to more than 55.0 wt.%, and the spherical shape of the alloys was preserved without sticking. By varying the monomer ratio, concentration fraction, and/or crosslinker type, the content of EPR in the iPP/EPR alloys can be controlled over a wide range. The component content of EPR and the gel content in the iPP/EPR alloys were determined via extraction using xylene. Thermal properties and crystallinity were measured and characterized through DSC and FTIR. The spectra are displayed in Supplementary Materials Figures S1 and S2. The results were calculated and are summarized in Table 2.

From the results, it is evident that the EPR content in the iPP/EPR alloys increased from 20.5 wt.% to 55.0 wt.%, with the gel content increasing to a moderate degree (~12.9 wt%). The DSC results show that the alloys displayed a typical melting temperature peak *T*_m_ (~162 °C), almost the same as that of iPP, which means that the thermal properties of the iPP/EPR alloys remain at a high level. With the increase in EPR, the alloys displayed a relatively low *T*_m_ for iPP/EPR-5 (i.e., 159.0 °C), which resulted from the high EPR content and crosslinking structure in the samples. Moreover, there was a small amount of crystallization at temperatures between 96.7 °C and 112.3 °C. With an increase in the EPR content, the major melt enthalpy decreased from 55.9 J g^−1^ to 27.0 J g^−1^, whereas the minor melt enthalpy displayed a small increase for iPP/EPR-4; this was due to the long ethylene crystalline segments generated during polymerization [31], which can be verified from the ^13^C NMR and FTIR results. On the one hand, the decreased crystallinity of crosslinked iPP/EPR is attributed to the large EPR content diluting the crystallization of iPP. On the other hand, this decrease is ascribed to the relatively high entanglement of the polymer chains and the longer time taken for the relaxation of the crosslinked EPR chains [32].

The microstructures were analyzed using ^13^C NMR measurement; the typical ^13^C NMR spectra of the iPP/EPR alloys and the soluble EPR are displayed in Figure S3. The sequence distribution of the iPP/EPR alloys and soluble EPR were calculated and are summarized in Table 3 and Table 4, respectively. From the ^13^C-NMR spectra, the chemical shifts (*δ*) at 46.3, 28.7, and 21.7 ppm belong to the characteristic carbon peaks of CH_2_, CH, and CH_3_, respectively. *δ* = 29.8 ppm is ascribed to the long ethylene segment. These peaks are displayed in the ^13^C-NMR spectra of the iPP/EPR alloys, i.e., *δ*: 37.7, 37.4, 33.1, 30.7, 30.2, 27.3, 20.5, and 19.8 ppm, which belong to random ethylene and propylene segment series. The contents of propylene (PP), ethylene–propylene (EP), and ethylene (EE) were tested and calculated using high-temperature ^13^C-NMR following the methods proposed by M. Kakugo [33]. The results are consistent with the extraction and FTIR results.

As per Table 3 and Table 4, the distribution of propylene and ethylene in iPP/EPR is closer to the content of EPR in iPP/EPR. However, the distribution of propylene and ethylene in soluble EPR and its sequence structure indicate random distribution. This also eventually resulted in the uniform distribution of the rubber phase and enhanced properties.

**Figure 2:**
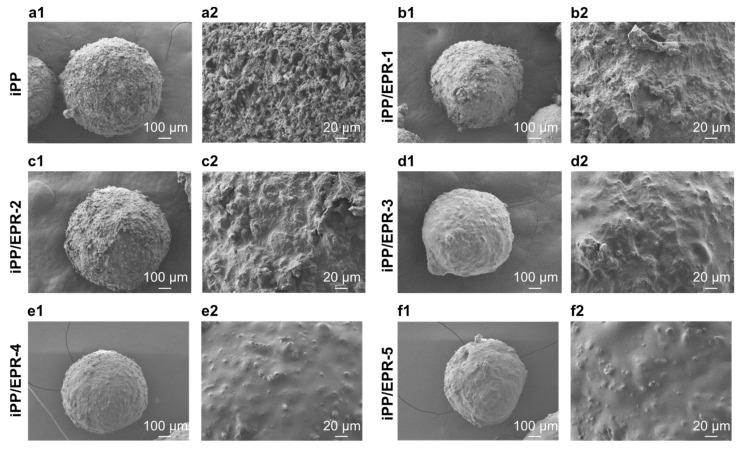
External phase morphologies of iPP and iPP/EPR alloys (**a1**–**f1**). Enlarged images (**a2**–**f2**).

The typical external morphologies of these iPP/EPR alloys were studied via SEM. As shown in Figure 2, the alloys have a granular shape and are different from each other. These in-reactor iPP/EPR alloys are about 1.0 mm in diameter, and secondary particles are clearly visible from the enlarged particle surfaces, which are about 100 μm in diameter. The surfaces of these alloys are clean and rough. For iPP and non-crosslinked iPP/EPR Figure 2(a1–b2), many primary iPP particles are present on the surface. The spherical shape and particle construction didn’t change a lot. With an increase in the EPR content and the crosslinking degree, the surface becomes smooth since there was much EPR filled in both the holes of the iPP primary particle voids and surfaces. Meanwhile, the surface of the alloys is still porous in iPP/EPR-2 (Figure 2(c1–c2)). This indicates that there are still some spaces in these alloys to allow the E/P gas monomers to flow into the internal spaces for copolymerization, leading to a high elastomer content. From the SEM images in Figure 2(a1–c2), it seen that the crosslinked iPP/EPR-2 (Figure 2(c1–c2)) still display a rough surface compared to pure iPP (Figure 2(a1–a2)), this is due to that there a little crosslinking in iPP/EPR-2 (*w_gel_* = 7.3%), and the EPR content still can be increased in the crosslinked iPP/EPR alloys. With the increase in the EPR content in the iPP/EPR alloys (Figure 2(b1–f2), the particles become compacted, and the surface becomes smooth. High-elastomer-content iPP/EPR alloys were obtained using in-reactor technology, and these alloys had good spherical morphology. The simultaneously crosslinked EPR chains during polymerization fixed part of the elastomer phase in the iPP matrix so that they did not stick in the kettle. It is interesting to find the effective generation of a branched/crosslinked structure during iPP multicomponent polymerization, efficiently hindering the fouling of EPR from the iPP particles.

**Figure 3:**
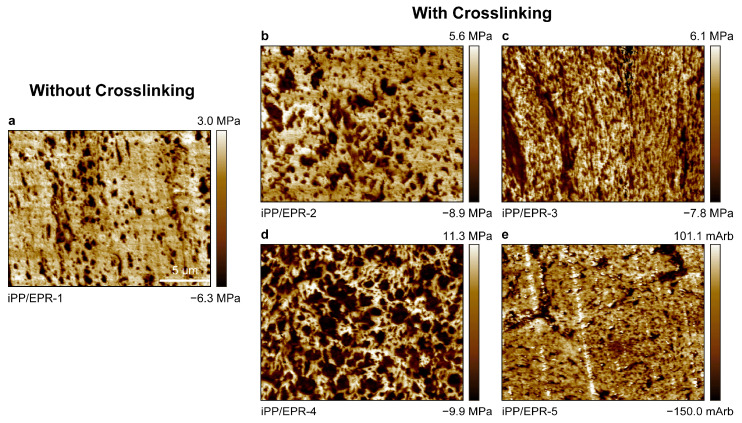
AFM images of the internal morphology of the iPP/EPR alloys without and with EPR chain crosslinking. The scale bar applied to these images is 5 μm (**a**–**e**).

The internal morphology and phase structure of the iPP/EPR alloys without and with crosslinked chain were observed via AFM. As shown in the images in Figure 3a, the EPR forms droplets and is randomly dispersed in the iPP matrix. In iPP/EPR-1, the EPR phase is relatively large, and the domain size is 0.5–1.0 μm. Most of the droplets are separately dispersed, and some of them are agglomerated and form a relatively large domain size of about 2.0 μm. Figure 3b–e show that the black EPR phase is finely dispersed and spreads throughout the matrix in crosslinked iPP/EPR with an EPR content of 55.0 wt.%. The EPR domains are less than 1 μm, which are much smaller than the plain iPP/EPR-1 alloys. With a moderate EPR content and crosslinking degree in iPP/EPR-3 (Figure 3c), i.e., *w_EPR_* = 35.0 wt.% and *w_gel_* = 7.3 wt.%, the EPR phase is gradually generated and finely dispersed in the matrix. As the rubber content in the alloys increases, the EPR phase becomes the majority phase in the alloys. When the EPR content further increases up to 50.6 and 55.0 wt.%, i.e., in iPP/EPR-4 and iPP/EPR-5 (Figure 3d,e), the EPR is still well-dispersed in the matrix as small droplets. The domain size is 20–100 nm.

The separately dispersed EPR indicates that the materials are not homogenous and display micro-phase separation for plain iPP/EPR. With simultaneous EPR crosslinking during copolymerization, the rubber phase was finely dispersed, and the domain became much smaller than those in the crosslinked iPP/EPR alloys. Through the simultaneous crosslinking of EPR chains in the iPP/EPR in-reactor alloys, the flow of the EPR phase was inhibited, so large-scale aggregation was impeded. In this case, macroscale phase separation was delayed during polymerization and processing. The results indicate the fine dispersion of EPR in the matrix. During EPR chain crosslinking, the EPR with a sufficient crosslinking degree adhered to the catalyst fragments to prevent the flow of the EPR phase from the inside of iPP to the macro-holes and/or alloys’ external surfaces. Under this circumstance, there were more interspaces and mesh holes for the ethylene and propylene monomers to synthesize EPR in the iPP particles, which were generated in the first homo-polymerization stage. Since non-conjugated α,ω-diene has two double bonds [30], it can be inserted in the E/P copolymerization stage. The terpolymers of propylene, ethylene, and diene further promote copolymerization reactions due to the high EPR content in iPP/EPR alloys.

**Figure 4:**
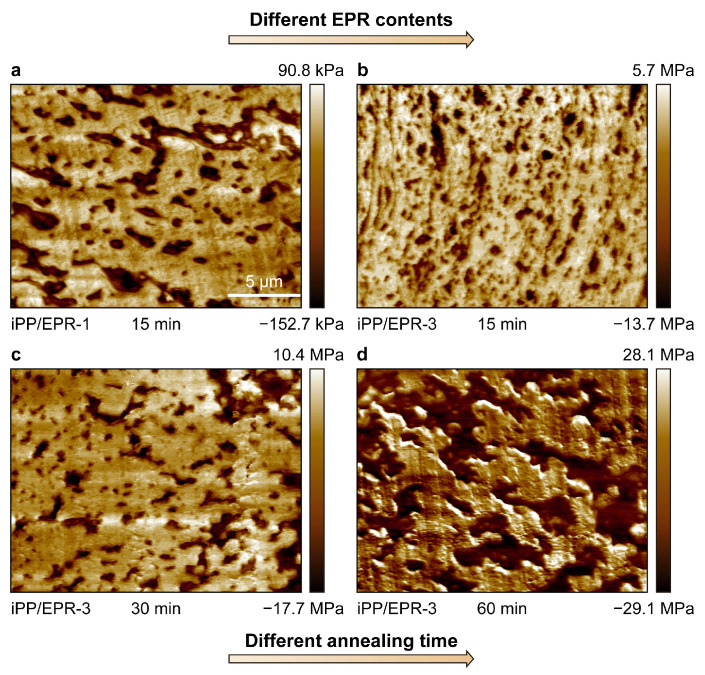
AFM images of morphology evolution of iPP/EPR alloys with different EPR contents (**a**,**b**) and different annealing time (**c**,**d**). The scale bar applied to these images is 5 μm (**a**–**d**).

Furthermore, the internal morphology and dispersion of EPR in the matrix after annealing were also observed through high-resolution AFM. The morphological evolution of the iPP/EPR alloys without and with chain crosslinking is displayed in Figure 4. In Figure 4a, the EPR content is 20.5 wt.% in iPP/EPR-1 without crosslinking. For iPP/EPR-3 in Figure 4b–d, the EPR content is 35.0 wt.%. In the iPP/EPR-3 alloys, minimally crosslinked EPR was generated by α,ω-diene, which was introduced into the reactor. After a relatively short annealing time of 15 min at 200 °C, the internal morphology was observed and is shown in Figure 4a,b. The black EPR domains in crosslinked iPP/EPR-3 are about 0.5–1.0 μm. The EPR domains in iPP/EPR-1 without crosslinking are about 2–3 μm, and some of them are slightly coarse. This indicates that the EPR domains are much smaller than those in iPP/EPR-1 after a relatively short annealing time of 15 min. The crosslinking of EPR in the alloys restricts the macroscopic coarsening of the EPR. Furthermore, with the annealing time being increased up to 30 min and 60 min for iPP/EPR-3, as shown in Figure 4c,d, the EPR domains become coarse and, finally, form a network after annealing for 60 min at 200 °C. This indicates that there was much more time for processing before the EPR phase became larger.

**Figure 5:**
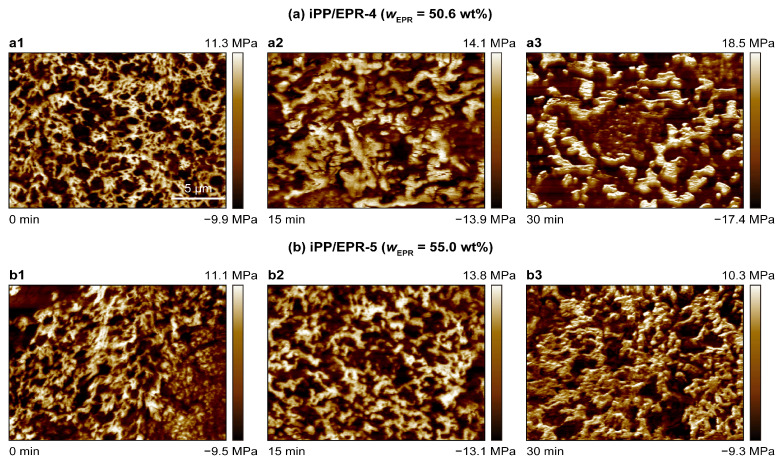
AFM images of morphology evolution of high EPR content of iPP/EPR alloys with chain crosslinking. Scale bar applied to these images is 5 μm (**a1**–**b3**).

For high-EPR-content iPP/EPR alloys, the internal morphology and dispersion of EPR in the samples after melt extrusion and annealing were also observed, and the AFM images are shown in Figure 5. In the upper row in Figure 5(a1–a3), the content of EPR in iPP/EPR-4 is 50.6 wt.%. In the lower row in Figure 5(b1–b3), the content of EPR in iPP/EPR-4 is 55.0 wt.%. It can be seen that the internal phase structure is very condensed. With an increase in the EPR content, EPR becomes the majority phase in iPP/EPR-4 and iPP/EPR-5. Due to chain crosslinking, the EPR phase in iPP/EPR-5 is smaller than that in iPP/EPR-4. However, in the highest-elastomer-content alloy iPP/EPR-5, i.e., *w_EPR_* = 55.0 wt.%, the EPR elastomer phase is present in small domains in the matrix. This is mainly due to the high crosslinking density of the alloys.

Based on the abovementioned results, the crosslinked iPP/EPR alloys were successfully obtained through the designed strategy, as shown in Figure 1. α,ω-diene took part in ethylene–propylene copolymerization, simultaneously inducing EPR chain branching/crosslinking. The increased EPR contents and finely dispersed EPR phase are consistent with the expectations. A highly branching and/or crosslinking EPR phase in in-reactor alloys will result in polymer entanglement and high elasticity.

### 3.2. Viscoelasticity of the Crosslinked iPP/EPR Alloys

**Figure 6:**
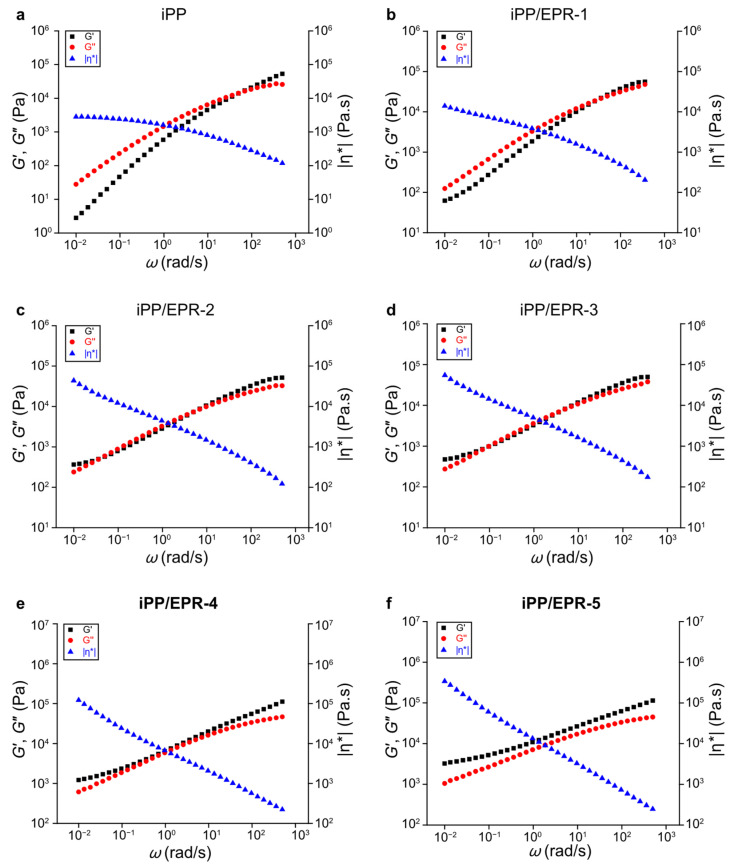

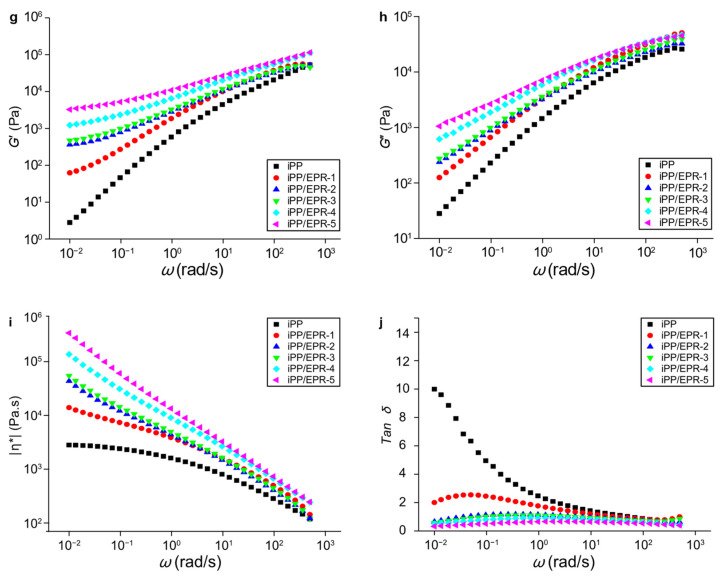
The dynamic frequency sweeps of iPP and iPP/EPR alloys. (**a**–**f**) represent the dynamic frequency sweeps of iPP, iPP/EPR-1, iPP/EPR-2, iPP/EPR-3, iPP/EPR-4, and iPP/EPR-5, respectively; (**g**–**j**) indicate the frequency dependence of *G*′, |η*|, and tan*δ* in the iPP and iPP/EPR alloys.

The viscoelasticity and rheological behavior of iPP/EPR in-reactor alloys can be easily tuned with chain crosslinking, especially when the elastomer content is high. As shown in Figure 6a,b, the storage (*G*′) and loss moduli (*G*″) of iPP and non-crosslinked iPP/EPR-1 are small in the low-frequency region (*ω*: 0.01–100 rad/s), which display liquid-like behavior. As shown in Figure 6c,d, there is a terminal platform in iPP/EPR-2 and iPP/EPR-3, suggesting that an elastic network was formed. For iPP/EPR-4 and iPP/EPR-5 in Figure 6e,f, with the increase in the EPR content and crosslinking degree, *G*′ diverges from *G*″, and there is no intersection in the dynamic frequency sweep for iPP/EPR-4 and iPP/EPR-5, which is a typical elastic response. This suggests that a highly entangled or crosslinked polymer chain network was formed [34,35]. The plateau in the terminal region obviously has a high EPR content and crosslinking degree (or gel content). This phenomenon is the result of the highly crosslinked EPR chains in the alloys. As per Figure 6g–j, *G*′ and |η*| are the lowest for iPP among all the studied samples. The *G*′ of iPP is below 10 Pa, and |η*| is below 1 × 10^2^ Pa.s at the lowest frequency (i.e., *ω* < 0.1 rad/s). The second-lowest one represents non-crosslinked iPP/EPR-1, with *G*′ < 100 Pa and |η*| < 1 × 10^4^ Pa.s. During the incorporation of diene for the EPR chain crosslinking of the iPP/EPR alloys, *G*′ and |η*| increased, and tan*δ* decreased correspondingly (Figure 6g–j). *G*′ is a reflection of the elastic characteristic of materials, and |η*| reflects the entanglement and mobility of polymer chains in rheology measurements [36]. According to the aforementioned rheological results, the elasticity was greatly enhanced in the crosslinked iPP/EPR alloys, and this is mainly attributed to the highly branched and/or crosslinked EPR chains. During the crosslinking of the EPR chains, polymer mobility was restricted, and the crosslinked iPP/EPR alloys became entangled, which also influenced the mechanical properties.

**Figure 7:**
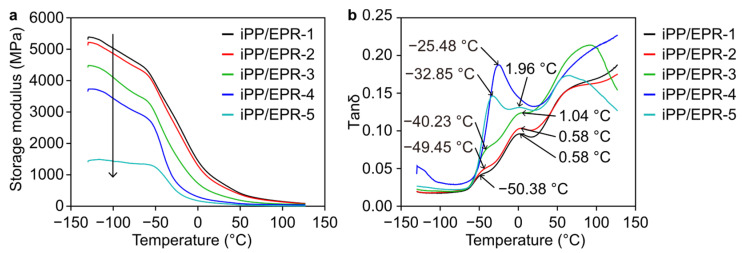
Temperature dependence of storage modulus (**a**) and tan*δ* (**b**) of crosslinked iPP/EPR alloys. *f* = 1 Hz.

The glass transition temperature (*T*_g_) can be verified according to the elastomer content and crosslinking degree. DMA measurements were used to determine the glass transition temperature (*T*_g_) and its variation (Δ*T*_g_); the storage modulus *E*′ and the damping parameter (tan*δ*) are presented in Figure 7a and 7b, respectively. *T*_g1_ and *T*_g2_, which correspond to the relaxation of EPR and iPP chain in the iPP/EPR alloys, respectively, and Δ*T*_g_ are summarized in Table 5. As seen in Figure 7a, the *E*′ of the iPP/EPE alloys decreases with increasing temperature. This is attributed to increased polymer chain mobility at high temperatures. From Figure 7b, in the damping parameter curves, *T*_g1_ and *T*_g2_ are present at around −45 °C and 1 °C. As the EPR content in the alloys increases, for iPP/EPR-1 and iPP/EPR-5, the two peaks become closer, ranging from 50.96 °C to 25.96 °C. The two peaks in Figure 7b are getting closer with the increase of EPR content. The smaller the value of Δ*T*_g_ in the alloys, which means the more compatible the components of iPP and EPR. The fine dispersion of EPR can also be seen from the phase structure in Figure 4 and Figure 5, as well as the ethylene and propylene sequence distribution in the NMR results. The results mean that crosslinked or highly branched EPR polymer chains, especially those that form an entanglement network, hinder the polymer chain diffusion and, hence, restrict the phase separation of the two components in these alloys [37,38]. In this case, the two components are more thoroughly mixed, and the final properties of these materials can be enhanced correspondingly.

### 3.3. Mechanical Properties of iPP and Crosslinked iPP/EPR Alloys

**Figure 8:**
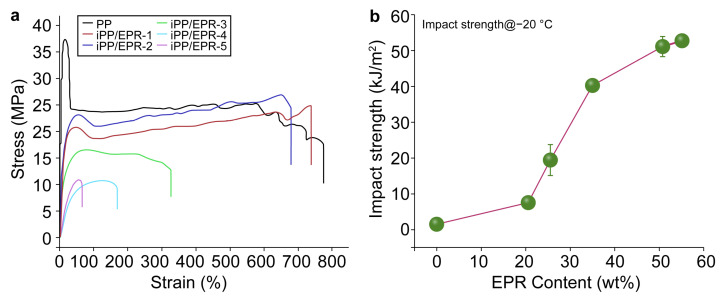
Mechanical properties of iPP and iPP/EPR alloys: (**a**) Stress-Strain curves and (**b**) Impact strength at low temperature (*T* = −20 °C).

By controlling the content and crosslinking degree of EPR in iPP/EPR alloys, the macroscopic mechanical properties of the materials can be tuned. Stress-Strain curves and impact strength at low temperature (*T* = −20 °C) were displayed in Figure 8a and 8b, respectively. The results of the tensile strength, tensile modulus, and impact properties as well as the elongation at break are summarized in Table 6. For iPP, the tensile and low-temperature impact strength are about 37.3 MPa and 1.4 kJ m^−2^, respectively. When EPR was crosslinked with diene during polymerization, the tensile strength decreased, while the impact strength obviously increased. Regarding the crosslinking of EPR in the alloys, for iPP/EPR-1 and iPP/EPR-6, the tensile strength decreased from 24.1 to 9.3 MPa compared to that of the non-crosslinked iPP/EPR alloys. Moreover, with an increase in the content of EPR, the tensile modulus and the elongation at break values further decrease from iPP to iPP/EPR-5, i.e., the tensile modulus decreases from 689 to 70.4 MPa, and the elongation at break decreases from 549% to 49.6%. Regarding the resistance to low temperatures, the impact strength increased from 7.4 kJ m^−2^ for iPP/EPR-1 to 44.0 kJ m^−2^ for iPP/EPR-5. For iPP/EPR-2, with some crosslinking in the alloys, the elongation at break and the impact strength at low temperatures were both enhanced. These enhanced mechanical properties are mostly related to the dispersion and phase structure of EPR in the alloys. Alloys with a network-like phase structure can achieve a good balance between strength and toughness. Additionally, the crosslinking of EPR restricts macroscopic phase separation and make processing more controllable. These properties are very useful in the automobile, construction, electronic, and electrical industries, such as for automobile bumpers, interiors, waterproofing rolls, and electric cable materials.

## 4. Conclusions

In summary, a series of iPP/EPR in-reactor alloys with a high EPR content were polymerized through the simultaneous crosslinking of EPR chains using non-conjugated α,ω-diene. Through the incorporation of a non-conjugated diene monomer in the copolymerization process, ethylene–propylene copolymer chains were randomly crosslinked. This can not only effectively control the rubber phase flow from iPP to avoid sticking in the kettle but also can effectively increase the EPR content of the rubber phase and control rubber’s fine dispersion in crosslinked iPP/EPR in-reactor alloys. Crosslinked EPR can effectively restrict the macro-phase separation of iPP and EPR in alloys. The rheological results show that the elasticity of iPP/EPR is enhanced with the crosslinking of EPR. Even with a small content of crosslinked EPR (less than 10 wt.%), elasticity can be increased by 2–3 times. iPP/EPR with a high EPR content shows elasticity and toughness. The increased EPR content reduces the tensile strength of the iPP/EPR in-reactor alloys, and the material evolves from plastic to elastic. In this case, the impact strength of the alloys is enhanced. The improved properties of the iPP/EPR alloys are mainly attributed to the increased EPR content and the compatibility of iPP and EPR, as well as the fine dispersion of EPR and the bi-continuous phase structure that had formed.

The development of high-elastomer-content iPP/EPR alloys using in-reactor technology with the simultaneous crosslinking of EPR chains using non-conjugated α,ω-diene monomers offers a novel approach to enhance the mechanical properties of these alloys. This approach not only addresses the issue of sticking during polymerization, but also improves the rubber phase distribution and elasticity, making it possible to achieve enhanced mechanical properties even at a low EPR content. The disappearance of the yielding peak during tensile testing further underscores the potential of these alloys for applications requiring enhanced impact strength and deformation behavior. This study provides valuable insights into the optimization of iPP/EPR alloys, potentially leading to advancements in various industrial applications.

## Figures and Tables

**Table 1:** Polymerization of the iPP and iPP/EPR alloys.

Samples	DHDCS [m mol]	Cat. [mg]	t1 [min]	t2 [min]	Yield [g]	Activity [g g_.cat_^−1^ h^−1^]	*w_EPR_* ^a^ [wt.%]
iPP	0	20.8	30	0	16.2	1560	0
iPP/EPR-1	0	22.0	30	16	22.5	1334	20.5
iPP/EPR-2	0.15	21.7	30	8	23.2	1688	25.4
iPP/EPR-3	0.30	22.8	30	60	46.2	1351	35.0
iPP/EPR-4	0.60	22.0	30	70	44.6	1216	50.6
iPP/EPR-5	1.35	22.3	30	40	59.2	2275	55.0

Conditions: *n*[TEA]/*n*[De] = 20:1; *n*[Al]:*n*[Ti] ≈ 200:1, Hexane: 50 mL; *T* = 60 °C. ^a^ Obtained from the soluble and insoluble parts in boiling xylene.

**Table 2:** Characteristic parameters of the crosslinked iPP/EPR alloys.

Samples	*w_EPR_* ^a^ [wt.%]	*w_gel_* ^a^ [wt.%]	*[E]* ^b^ in EPR [mol%]	*T*_c_ ^c^ [°C]	Δ*H*_c_ ^c^ [J g^−1^]	*T*_m_ ^c^ [°C]	Δ*H*_m_ ^c^ [J g^−1^]
iPP	0	0	0	112.5	96.6	159.9	75.2
iPP/EPR-1	20.5	0	40.0	112.3;102.5	0.2;60.3	117.3;163.2	1.6;55.9
iPP/EPR-2	25.4	7.3	40.2	109.0	64.6	117.3;163.2	1.6;55.8
iPP/EPR-3	35.0	7.3	40.7	117.5	54.5	117.1;162.2	1.8;43.4
iPP/EPR-4	50.6	8.9	38.7	96.7;109.1	0.28;20.7	116.7;161.4	3.1;23.6
iPP/EPR-5	55.0	12.9	37.0	96.7;112.0	0.4;26.0	113.1;159.0	1.5;27.0

^a^ Obtained from the soluble and insoluble parts in boiling xylene; ^b^ calculated from FTIR analysis, *[E]* = [1.236 − 1.575(A_1376_/A_1459_)] × 100%, *[E]* + [P] = 100%; ^c^ data obtained from the second heating scan of DSC measurement.

**Table 3:** Sequence distribution of iPP/EPR alloys.

Samples	P	E	PP	PE	EE	PPP	PPE	EPE	PEP	EEP	EEE	*l_P_*	*l_E_*
iPP/EPR-1	79.29	20.71	72.99	12.62	14.40	66.30	8.14	2.86	3.72	6.54	12.43	12.57	3.28
iPP/EPR-2	74.36	25.64	66.60	15.51	17.88	60.53	8.79	3.29	4.51	7.98	14.89	9.59	3.31
iPP/EPR-3	65.55	34.45	53.51	24.07	22.41	44.54	13.37	5.07	6.34	12.48	18.21	5.45	2.86
iPP/EPR-4	55.46	44.54	40.48	29.96	29.56	30.08	17.47	6.60	7.50	13.35	21.33	3.70	2.97
iPP/EPR-5	54.35	45.65	39.08	30.54	30.38	31.49	16.93	7.43	7.43	14.70	22.02	3.56	2.99

**Table 4:** Sequence distribution of soluble EPR.

Samples	P	E	PP	PE	EE	PPP	PPE	EPE	PEP	EEP	EEE	*l_P_*	*l_E_*
EPR-1	59.10	40.90	41.03	36.13	22.84	29.40	19.03	7.20	8.57	20.23	15.56	3.27	2.26
EPR-2	57.90	42.10	39.10	37.60	23.30	28.54	20.03	7.41	10.01	17.72	16.30	3.08	2.24
EPR-3	57.73	42.27	37.86	39.75	22.39	26.56	21.64	6.87	10.25	19.89	14.79	2.90	2.13
EPR-4	55.51	44.49	35.23	40.56	24.21	25.38	23.28	8.87	11.08	17.07	14.33	2.74	2.19
EPR-5	54.19	45.81	33.74	40.89	25.36	24.42	21.89	10.11	10.53	17.89	15.16	2.65	2.24

**Table 5:** Glass transition temperature of iPP and crosslinked iPP/EPR alloys.

Samples	*w_EPR_* [wt.%]	*T*_g1_ [°C]	*T*_g2_ [°C]	Δ*T*_g_ [°C]
iPP	0	/	10.8	/
iPP/EPR-1	20.5	−50.38	0.58	50.96
iPP/EPR-2	25.4	−49.45	0.58	50.03
iPP/EPR-3	35.0	−40.23	1.04	41.27
iPP/EPR-4	50.6	−32.85	1.96	34.81
iPP/EPR-5	55.0	−25.48	0.48	25.96

**Table 6:** Mechanical properties of crosslinked iPP/EPR alloys.

Samples	*w_EPR_* [wt.%]	*w_gel_* [wt.%]	Tensile Strength [MPa]	Tensile Modulus [MPa]	Elongation at Break [%]	Impact Strength [kJ m^−2^] at −20 °C
iPP	0	0	37.3 ± 0.47	689.0 ± 33.9	549 ± 318.5	1.4 ± 0.01(C)
iPP/EPR-1	20.5	0	24.1 ± 1.2	308.1 ± 28.7	715.2 ± 33.9	7.4 ± 0.25(C)
iPP/EPR-2	25.4	7.3	26.4 ± 0.7	287.4 ± 1.2	715.4 ± 50.7	19.1 ± 4.3(P)
iPP/EPR-3	35.0	7.3	16.5 ± 0.05	151.4 ± 30.4	396.1 ± 98.1	40.2 ± 1.8(P)
iPP/EPR-4	50.6	8.9	11.3 ± 0.95	46.5 ± 6.8	130.0 ± 55.4	35.2 ± 8.5(P)
iPP/EPR-5	55.0	12.9	9.3 ± 2.2	70.4 ± 6.5	49.6 ± 23.0	44.0 ± 2.1(P)

(C) Completely broken; (P) unbroken specimen.

## Data Availability

Data are contained within the article and Supplementary Materials.

## Supplementary Materials

The following supporting information can be downloaded at https://www.mdpi.com/article/10.3390/polym16192715/s1, Figure S1: DSC Cooling (a) and reheating (b) curves of iPP and iPP/EPR alloys; Figure S2: FTIR spectra of soluble EPR in the iPP/EPR alloys; Figure S3: Typical 13C NMR spectra of the iPP/EPR alloys and the soluble EPR. (a) iPP/EPR-3, and (b) EPR-3, respectively.
