# Supplementary material for: Effect of Phase Structure on the Viscoelasticity and Mechanical Properties of Isotactic Polypropylene Multicomponents Polymerized with Non-Conjugated α,ω-Diene
Source: Polymers (Basel). 2024 Sep 25;16(19):2715. doi: 10.3390/polym16192715 (PMC11479073; doi:10.3390/polym16192715)
Supplement: Supplementary file 1 [file polymers-16-02715-s001.zip › polymers-3182606-supplementary.pdf]

Supplementary Materials

Effect of Phase Structure on the Viscoelasticity and Mechanical Properties of Isotactic Polypropylene Multicomponent Polymerized with Non-conjugated α,ω-Diene

Songmei Zhao [a,*], Jin-Yong Dong [b,c], Yawei Qin [b], Chuanzhuang Zhao [d], Yuan Yu [a] and Weili Liu [a,*]

[a] Institute of New Materials and Advanced Manufacturing, Beijing Academy of Science and Technology, Beijing, China, 100089
[b] CAS Key Laboratory of Engineering Plastics, Institute of Chemistry, Chinese Academy of Sciences, Beijing, China, 100190
[c] University of Chinese Academy of Sciences, Beijing, China, 100049
[d] School of Materials Science & Chemical Engineering, Key Laboratory of Impact and Safety Engineering, Ministry of Education, Ningbo University, Ningbo, China, 315211
* Correspondence: whom correspondence should be addressed
E-mail: zhaosongmei@bjast.ac.cn(S.Z.); liuweili@iccas.ac.cn(W.L.)
Tel.: +86 10 66020472(S.Z.); Fax: +86 10 66005741(W.L.).

Citation: Zhao, S.; Dong b, J.-Y.; Zhao d, C.; Yu, Y.; Liu a, W. Effect of Phase Structure on the Viscoelasticity and Mechanical Properties of Isotactic Polypropylene Multicomponent Polymerized with Non-conjugated α,ω-Diene. **2024**, *16*, x. https://doi.org/10.3390/xxxxx

Academic Editor(s): Name

Received: date
Revised: date
Accepted: date
Published: date

Copyright: © 2024 by the authors. Submitted for possible open access publication under the terms and conditions of the Creative Commons Attribution (CC BY) license (https://creativecommons.org/licenses/by/4.0/).

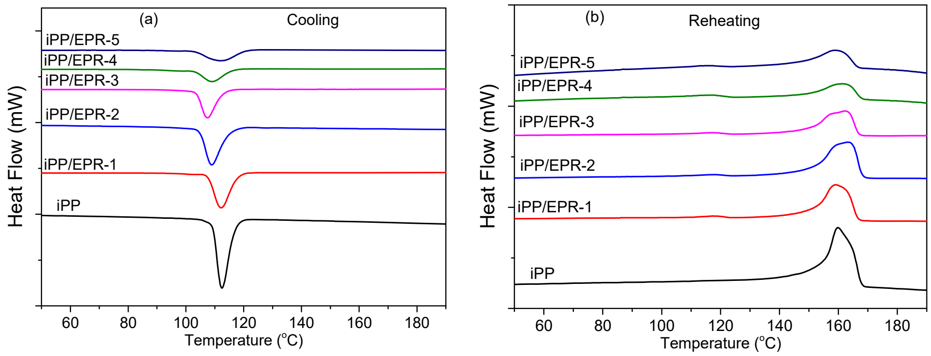

Figure S1. DSC Cooling (a) and reheating (b) curves of iPP and iPP/EPR alloys.

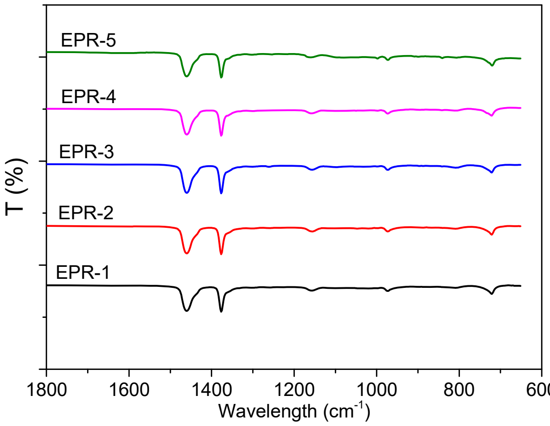

Figure S2. FTIR spectra of soluble EPR in the iPP/EPR alloys.

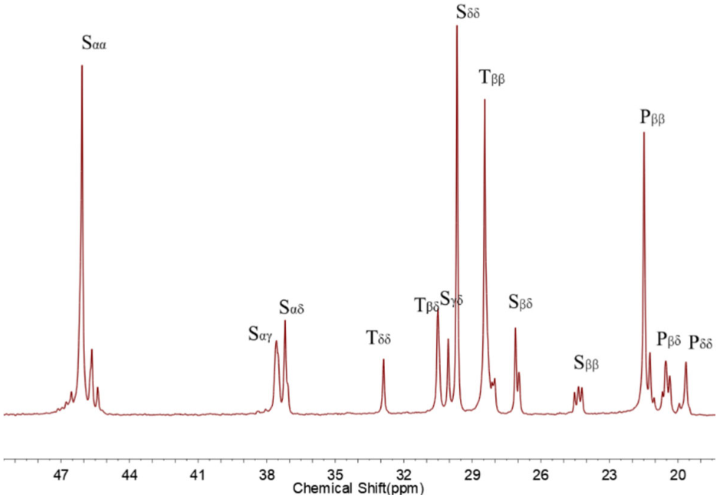

(a)

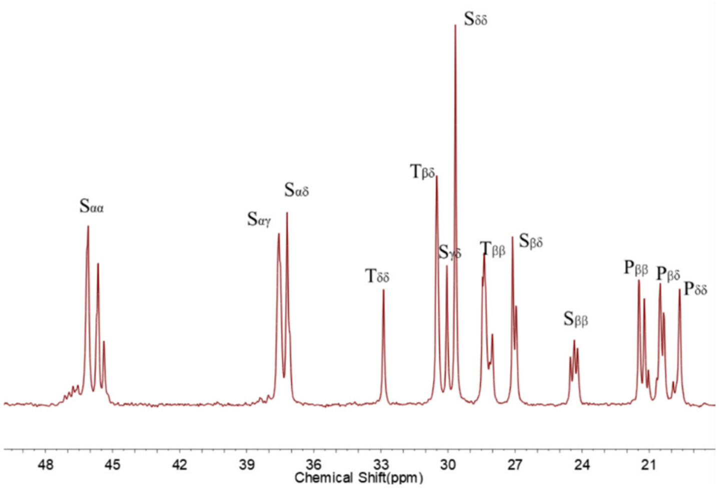

(b)

Figure S3. Typical 13C NMR spectra of the iPP/EPR alloys and the soluble EPR: (a) iPP/EPR-3, and (b) EPR-3, respectively.

The primary, secondary, and tertiary carbon atoms in the copolymer are used, respectively. The letters P, S, and T are indicated, and the two Greek letters below represent the distance between the analyzed carbon atom and the closest tertiary carbon atom on the molecular chain. The sequence distributions were calculated according to the following formulas [1]:

The methylene peak in the 13C NMR spectrum can be used to obtain the distribution of binary groups in the polymer:

$$PP = S_{\alpha\alpha}$$

$$PE = S_{\alpha\gamma} + S_{\alpha\delta}$$

$$EE = \frac{1}{2}(S_{\beta\delta} + S_{\delta\delta}) + \frac{1}{4}S_{\gamma\delta}$$

The distribution of three unit groups in the polymer can be obtained based on the methylene peak and the methylene peak.

$$PPP = T_{\beta\beta}$$

$$PPE = T_{\beta\delta}$$

$$EPE = T_{\delta\delta}$$

$$PEP = S_{\beta\beta} = 1/2 S_{\alpha\gamma}$$

$$EEP = S_{\alpha\delta} = S_{\beta\delta}$$

$$EEE = 1/2\ S_{\delta\delta} + 1/4 S_{\beta\delta}$$

The composition of propylene monomer and ethylene monomer in polymers can be calculated through the distribution of two unit groups and three unit groups.

$$P = PP + \frac{1}{2}PE$$

$$E = EE + \frac{1}{2}PE$$

$$P = PPP + PPE + EPE$$

The sequences were calculated from the 13C NMR spectra of the iPP/EPR alloys and the soluble EPR and summarized in the manuscript.

References

1. Kakugo, M.; Naito, Y.; Mizunuma, K.; Miyatake, T. Carbon-13 NMR determination of monomer sequence distribution in ethylene-propylene copolymers prepared with δ-titanium trichloride-diethylaluminum chloride. *Macromolecules* **1982**, *15*, 1150–1152.
